# The Long Term Role of Anxiety Sensitivity and Experiential Avoidance on Pain Intensity, Mood, and Disability among Individuals in a Specialist Pain Clinic

**DOI:** 10.1155/2016/6954896

**Published:** 2016-03-07

**Authors:** S. Mehta, D. Rice, S. Janzen, J. Serrato, H. Getty, A. P. Shapiro, P. Morley-Forster, K. Sequeira, R. W. Teasell

**Affiliations:** ^1^Western University, London, ON, Canada; ^2^St. Joseph's Health Care London, London, ON, Canada; ^3^Lawson Health Research Institute, London, ON, Canada

## Abstract

*Background.* Anxiety sensitivity (AS) and experiential avoidance (EA) have been shown to have an interactive effect on the response an individual has to chronic pain (CP) potentially resulting in long term negative outcomes.* Objective.* The current study attempted to (1) identify distinct CP subgroups based on their level of EA and AS and (2) compare the subgroups in terms of mood and disability.* Methods.* Individuals with CP were recruited from an academic pain clinic. Individuals were assessed for demographic, psychosocial, and personality measures at baseline and 1-year follow-up. A cluster analysis was conducted to identify distinct subgroups of patients based on their level of EA and AS. Differences in clinical outcomes were compared using the Repeated Measures MANOVA.* Results.* From a total of 229 participants, five clusters were formed. Subgroups with lower levels of AS but similar high levels of EA did not differ in outcomes. Mood impairment was significantly greater among those with high levels of EA compared to lower levels (*p* < 0.05). Significant improvement in disability (*p* < 0.05) was only seen among those with lower levels of EA and AS.* Conclusions.* This cluster analysis demonstrated that EA had a greater influence on mood impairment, while both EA and AS levels affected disability outcomes among individuals with CP.

## 1. Introduction

Anxiety plays a significant role in a patient's response to pain. A key concept of anxiety is anxiety sensitivity (AS), which is defined as the fear of anxiety-related sensations; specifically, fear of bodily sensations due to beliefs that these sensations will have a negative somatic, cognitive, or social consequence [1]. AS has been included in the fear-avoidance model as a vulnerability variable which could explain individual differences in fear of pain [2]. As individuals attempt to increase control or avoid negatively evaluated experiences, this often results in increased psychological distress and the use of poor coping strategies.

Coping strategies are important and are often described in terms of adaptive and maladaptive styles. The role of adaptive coping styles is to promote a sense of self-control within the patient in response to negative thoughts, behaviours, and feelings [3]. AS may result in maladaptive coping among individuals with chronic pain, leading to negative outcomes. For some patients the daily struggles of chronic pain become overwhelming, especially if they have high levels of AS. This can compel individuals to present experiential avoidant behavior and become self-protective and avoidant of potential threats related to pain. Experiential avoidance (EA) is a process that involves excessive negative evaluations, whereby persons avoid upsetting emotions, thoughts, feelings, and bodily sensations [4, 5]. Though the interaction between AS and EA may temporarily alleviate distress, it may exacerbate long term negative outcomes [6]. Individuals that are less likely to be in contact with unwanted experiences and are more likely to try to control sensations have been shown to have decreased functioning and increased distress [7].

The present study hypothesized that AS and EA play a highly influential role on mood and distress, pain disability, and pain intensity amongst patients with chronic pain. As such, this study sought to (1) divide individuals with chronic pain into subgroups according to their level of EA and AS and (2) identify which subgroups of patients with pain are most at risk to experience these comorbidities.

## 2. Method

### 2.1. Participants and Procedure

Participants were recruited from an academic specialist pain outpatient clinic in London, ON. Participants had to meet the following inclusion criteria: be admitted to the outpatient pain clinic, have a diagnosis of chronic pain (≥3 months), and be between the ages of 18 and 65. Given that this study involved the completion of self-report questionnaires, patients with an inability to read and write in English were excluded.

Patients who met the inclusion criteria were mailed a questionnaire booklet and consent form two weeks prior to their scheduled appointment at the pain clinic. Subsequently, the research assistant contacted the participants by telephone to answer any questions and instruct participants to complete the booklet prior to their appointment. The booklet consisted of background information related to the patient's demographic characteristics and the following questionnaires: Anxiety Sensitivity Index (ASI), Acceptance to Action Questionnaire (AAQ), average pain intensity, Depression Anxiety Stress Scales-Short Form (DASS-SF), and Pain Disability Index (PDI). The battery of questionnaires administered to patients was solely used for research purposes. The questionnaires were not seen by the treating physician and thus did not impact patients' treatment for chronic pain. Participants were asked to arrive half an hour before their appointment was scheduled to begin. Upon arrival at the clinic, the research assistant collected the questionnaire booklet and made sure to clarify with the participant that any answers left blank were purposely skipped. Subjects were mailed a follow-up questionnaire booklet 12 months following the in-person assessment, which included average pain intensity, DASS-SF, and PDI. Similar to the original protocol, contact was made with the participant via telephone to answer any questions and encourage timely completion. A self-addressed, stamped, return envelope was provided. Subjects who did not return their questionnaire booklet following three postcard reminders received a subsequent telephone reminder. All procedures were approved by the University of Western Ontario Health Sciences Review Board.

### 2.2. Cluster Variable Measures

#### 2.2.1. Anxiety Sensitivity Index

The ASI [8] is a 16-item measure of the fear of anxiety-related symptoms comprised of three factors: fear of the somatic symptoms of anxiety, fear of mental incapacitation (“cognitive dyscontrol”), and fear of negative social repercussions of anxiety [9]. Each item is rated on a five-point Likert scale ranging from 0 (very little) to 4 (very much). The instrument's psychometric properties and predictive validity have been well established [8, 10].

#### 2.2.2. Acceptance and Action Questionnaire

The AAQ [11] is a 9-item self-report measure of EA or the unwillingness to remain in contact with distressing private experiences (body sensations, emotions, and thoughts) and the inclination to alter the form or frequency of these experiences. Respondents rate the degree to which items apply to them on a 7-point scale ranging from 1 (“never true”) to 7 (“always true”). It yields a single factor solution with higher scores indicating increased avoidance and immobility. Higher scores on the AAQ have been found to correlate with a wide range of negative behavioural and physical health outcomes [11]. The AAQ demonstrates adequate validity and reliability scores [11].

### 2.3. Outcome Measures

#### 2.3.1. Average Pain Intensity

Individuals were asked to rate their average pain over the past two weeks on an 11-point numeric rating scale, ranging from 0 to 10, with 0 being no pain and 10 being worst pain. This composite pain intensity score has been shown to be a very reliable measure of pain intensity in chronic pain patients and has been used in recent research [6].

#### 2.3.2. Depression Anxiety Stress Scales-Short Form

The DASS-SF [12] is a 21-item self-report measure assessing depression, anxiety, and stress over the previous week. This short form scale is an abbreviated version of the 42-item scale developed by P. F. Lovibond and S. H. Lovibond [12] and presents the same established psychometric properties as the original scale [13]. The items are scored on a 4-point scale (0 = “did not apply to me at all” to 3 = “applied to me very much or most of the time”); higher scores indicate greater levels of distress.

#### 2.3.3. Pain Disability Index

The PDI [14] is a 7-item questionnaire asking participants to rate the degree to which pain interferes with functioning across seven domains: family/home responsibilities, recreation, social activity, occupation, self-care, life support activity, and sexual functioning. Participants score each item on a scale from 0 (“no disability”) to 10 (“worst disability”). Scales are summed to derive a total disability index. In this study, the sexual functioning item was omitted with the goal of minimizing missing data [15].

### 2.4. Data Analysis

A two-step cluster analysis was performed to organize observations into two or more mutually exclusive groups, where members of the group share properties in common. The two-step cluster analysis procedure (i.e., hierarchical and *k*-means analyses) provides the maximum flexibility in determining the appropriate number of clusters. This hierarchical approach identifies the optimal number of clusters that maximizes differences between clusters while minimizing within-group differences on the dependent variables [16]. This process can also involve visually inspecting the “kink,” or breakpoint in the plot, where the natural number of clusters is exceeded. When the number of clusters increases, the mean distance between the elements in the cluster declines at the “kink” in the plot, representing the number of clusters where the mean distance is not much higher than when one more cluster is assumed. The *k*-means nonhierarchical procedure then confirms the number of clusters identified. This method of clustering provides a robust identification of clusters and capitalizes on the strengths of both methods while compensating for weaknesses [17, 18].

Two clustering variables were used in the analysis: AS and EA measured by the ASI and AAQ, respectively. The log-likelihood distance measure was used, with subjects assigned to the cluster leading to the largest likelihood. The number of clusters was not predetermined. The Bayesian information criterion was used to judge adequacy of the final solution. Differences in sample demographic characteristics were compared according to cluster membership using univariate analysis of variance for continuous variables and *χ* ^2^ tests for categorical variables in order to characterize the resulting clusters. A repeated measures MANOVA was conducted on continuous outcome measures (pain intensity, DASS-SF total, DASS-SF depression, DASS-SF anxiety, DASS-SF stress, and pain disability) according to cluster membership to compare patient scores at baseline and one-year follow-up. Post hoc analyses were conducted with a Tukey correction to compare mood, pain intensity, and pain disability between cluster differences. All tests were performed using SPSS version 23.0 (Chicago, IL) and were two-tailed. The significance level was set at 0.05.

## 3. Results

In total 383 patients were eligible to participate during the study period, 229 agreed to participate in the study and completed study questionnaires. The study population had an average age of 45.6 years (SD = 11.5) and comprised predominantly females (64.2%). The majority of individuals were married or in a serious relationship (73.1%) and had experienced chronic pain for less than 10 years (84.8%).

**Figure 1:**
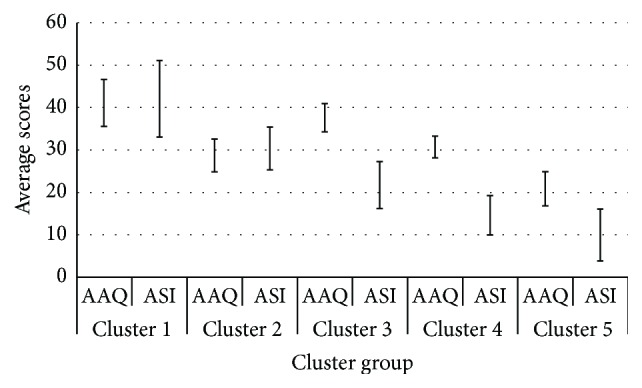
Two-step cluster subgroups based on Acceptance and Action Questionnaire and Anxiety Sensitivity Index.* Note*: AAQ: Acceptance to Action Questionnaire; ASI: Anxiety Sensitivity Index (ASI).

The results from the bivariate correlational analyses at baseline are shown in Table 1. The two-step cluster analysis divided participants into five clusters based on levels of AS and EA. Inspection of the Bayesian information criterion confirmed that the obvious “kink” in the plot was at the five-cluster solution [19]. Both AAQ and ASI were significantly different among the five clusters (*p* < 0.001).  Table 2 reports on the study and demographic characteristics of the 5 clusters. Cluster 1 (*n* = 38) had the highest levels of both EA and AS; these individuals constitute patients with the highest levels of both AS and EA. Participants in Cluster 2 (*n* = 26) scored the second highest for AS but the second lowest for EA; these individuals could be characterized as those that were functional copers with high anxiety sensitivity. AS scores continually decreased in Clusters 3 (*n* = 59), 4 (*n* = 63), and 5 (*n* = 43), respectively. The EA scores for Clusters 3, 4, and 5 followed a similar pattern; however, Cluster 3 scored above the mean EA scores while Clusters 4 and 5 scored below the mean (Table 2 and Figure 1). Those in Cluster 3 could be characterized as scoring average on both AS and EA. Individuals in this cluster may be considered “average copers” of chronic pain. Cluster 4 individuals had average levels of EA and low levels of AS. These may constitute a subgroup of moderately avoidant individuals with low sensitivity. Lastly, those in Cluster 5 could be characterized as those with very low EA and AS, and hence these individuals may be effective adaptive copers. Demographic characteristics (age, sex, relationship status, and pain duration) did not significantly differ among clusters (Table 2).

### 3.1. Effect of Time

Repeated measures analysis with Tukey correction resulted in an overall improvement of average pain intensity scores within the full sample of patients when comparing baseline to follow-up scores, representing a main effect of time on average pain intensity (*F*(1, 223) = 9.52, *p* < 0.006). However, no significant main effect for time (within-subject) was found on mood (DASS-SF scales) or disability (PDI) scores from baseline to follow-up. Additionally, no significant interactions between time and clusters on average pain intensity (*F*(4,223) = 0.74, *p* = 0.056), depression (*F*(4,223) = 0.96, *p* = 0.43), anxiety (*F*(4,223) = 1.05, *p* = 0.38), stress (*F*(4,223) = 18.02, *p* = 0.26), or disability (*F*(4,223) = 3.35, *p* = 0.11) were found based on the repeated measures MANOVA.

### 3.2. Effect of Cluster Subgroups

As seen in Table 3, when considering individual clusters, there was a significant main effect (between subject) of clusters for DASS-SF total (*F*(4, 221) = 11.81, *p* < 0.001), DASS-SF depression (*F*(4, 223) = 10.76, *p* < 0.001), DASS-SF anxiety (*F*(4, 221) = 10.62, *p* < 0.001), DASS-SF stress (*F*(4, 223) = 12.28, *p* < 0.001), and PDI scores (*F*(4, 223) = 4.83, *p* < 0.001). No significant main effect (between subject) of cluster was seen in average pain intensity (*F*(4,223) = 1.45, *p* = 0.22).

Post hoc analysis resulted in a number of significant differences among clusters on mood and pain disability scores (Table 4). Cluster 1 scored significantly higher than Clusters 2, 4, and 5 on DASS-SF total, DASS-SF depression, DASS-SF anxiety, and DASS-SF stress while also scoring significantly higher than Cluster 5 for pain disability (all *p* < 0.05). Cluster 3 also scored significantly higher than Clusters 4 and 5 on DASS-SF total, DASS-SF depression, DASS-SF anxiety, and DASS-SF stress and scored significantly higher on pain disability compared to Cluster 5 (all *p* < 0.05). Cluster 2 did not significantly differ from any of the other clusters.

## 4. Discussion

To our knowledge the current study is the first to subdivide individuals with a diagnosis of chronic pain based on their levels of EA and AS and compare the subgroups on outcomes of pain intensity, mood, and disability over time. Five clusters emerged within our sample when patients were grouped based on levels of EA and AS. Overall, average pain intensity decreased from baseline to one year follow-up, while no significant difference in pain intensity was seen between clusters. Hence, it can be assumed that the changes in distress and disability are not necessarily related to a differential in pain intensity among the different subclusters.

No significant difference in mood and disability was seen overall between scores at baseline and follow-up; however, when participants were subdivided into five subgroups based on their level of EA and AS, significant differences were found among the clusters for mood and disability after one year. Consistent with our hypothesis, Cluster 1, individuals with the highest levels of EA and AS, experienced greater levels of distressed mood and disability compared to those in Cluster 5 who reported low levels of EA and AS. A previous cross-sectional study by Esteve and Ramírez-Maestre [20] also found that individuals with high levels of EA and AS had more negative moods. The increased levels of EA and AS lead to a greater unwillingness to endure upsetting emotions, thoughts, memories, and other private experiences that lead to maladaptive efforts to resist, escape, and avoid these experiences [21]. This may have important implications when examining responsiveness to treatment. Individuals with high levels of AS and EA may be more resistant to single modal treatments and require a more comprehensive multidisciplinary protocol.

Individuals in Cluster 2 who reported moderate levels of both EA and AS did not differ from those in Cluster 3, 4, or 5 who had lower levels of mood and disability. However, the individuals in Cluster 2 had significantly lower levels of distress and disability compared to Cluster 1 (highest levels of EA and AS). This finding may indicate that there is a threshold of EA and AS levels required for perceiving greater disability and distress. Those with moderate levels of EA and AS appear to cope as well as the patients in Clusters 3, 4, and 5, which reported the lowest EA and AS levels. This suggests that experiencing a moderate amount of inflexibility and need for control may not substantially reduce the coping capability of patients with chronic pain.

Surprisingly, individuals from both Clusters 1 and 3 significantly differed from Cluster 5 on their reports of pain disability. Individuals in these two subgroups had similarly high levels of EA; however, those in Cluster 3 were almost 2 standard deviations below Cluster 1 in AS scores and were only 1 standard deviation above Cluster 5 scores. This finding suggests that pain disability may be independently influenced by levels of EA, rather than AS, among individuals with chronic pain. This is further strengthened by the finding that although Cluster 2 included individuals with higher AS levels than those in Cluster 3 (1 standard deviation above), their moderate levels of EA did not result in significant differences in mood and disability compared to Cluster 5. Esteve et al. [22] examined the relationship between EA and AS in adjusting to chronic pain. This study found that AS was moderately associated with depression and anxiety, while EA was only weakly correlated. Contrary to the current study, Esteve et al. [22] found that AS had a stronger association than EA with pain fear-avoidance. Although EA and AS are both associated with negative outcomes, the current study demonstrates that EA may have a greater influence on mood and disability, as it involves the avoidance of negative experiences and situations rather than just anxiety-sensations. These results are consistent with a study that compared subgroups of patients diagnosed with rheumatoid arthritis based on their EA and AS levels [23]. In the study, individuals in the lowest levels of EA and AS reported significantly improved levels of mood, disability, and quality of life compared to those in the high or moderate groups [23]. McCracken and Keogh [24] found that acceptance and mindfulness based therapy that is designed to reduce EA was able to decrease the effect of AS on a patients' ability to function but did not eliminate this effect. The use of multimodal treatment strategies may be necessary for optimal management of these individuals. Treatments strategies that target the physiological, cognitive, and emotional aspects of anxiety that patients with chronic pain may face are necessary to positively influence patients' pain management and help alleviate associated distress. Teaching coping strategies through acceptance and commitment therapy which targets acceptance and willingness to experience unwanted events may be beneficial for these high risk individuals [25]. Other strategies such as cognitive behavior therapy, which uses mindfulness approaches to help an individual develop more cognitive flexibility and distress tolerance, may also be applied to decrease anxiety symptoms that patients with chronic pain experience [26]. Numerous studies have shown that acceptance of pain is associated with less disability and distress and decreased use of health care resources [27, 28].

Consistent with other studies examining levels of AS among individuals with chronic pain, the current study found that patients reported significantly greater levels of AS than the general population. Overall, the current study population had ASI scores 1 standard deviation above the general population mean based on normative data (mean = 19.01; standard deviation = 9.11) collected by Pollard [14]. Individuals in Clusters 1–3 were at least 1 standard deviation above the normative mean, and Cluster 4 was 2 standard deviations above the normative mean, while only Cluster 5 scores were close to the average. Levels of EA among individuals in our study were lower than reports collected from undergraduate students [15].

The current study had several limitations. First, our population consisted of patients treated at a specialist pain clinic. Previous studies have shown that these individuals may have longer histories of pain, have undergone more interventions, and consume the highest amounts of pain medications [20]. Hence, these individuals may already be at a heightened risk for secondary pain conditions when compared to those not referred to a specialist pain clinic. Second, the type and frequency of treatment that participants received were not recorded. This information may have influenced the outcomes discussed in the paper. However, since overall pain intensity decreased and there was no significant difference in pain intensity among the clusters, it is not unreasonable to assume that all participants received treatments or support which resulted in an overall decrease in average pain. Additionally, it should be considered that cluster analysis partially relies on the interpretability of subgroups and the ability of these groups to be meaningfully labelled and described, which can be a subjective task.

Assessment of EA and AS was conducted only at baseline; however, it may be beneficial to assess if there is an effect of time on both measures in future studies. Evaluating the stability of these trait-like constructs over time among individuals with chronic pain may be of importance. Rosellini et al. [29] found that the temporal course of AS was similar to that of depression after various treatments. Furthermore, examining change in EA and AS over a period of time, especially in conjunction with improvement of pain intensity, would help to understand the directional relationship between the two.

Despite these limitations, the current study has important research and clinical implications. This study has expanded our understanding of the effect of AS and EA on mood and disability among individuals with chronic pain. Further, it has demonstrated that individuals with high levels of EA and AS are at risk for experiencing negative mood and greater disability. The study found that EA, rather than AS, may be related to disability among those with chronic pain. Identification of these individuals may help clinicians develop multimodal management plans resulting in more optimal outcomes. Use of acceptance based strategies and cognitive behavioural therapy which help individuals improve their willingness to experience unwanted events and improve their cognitive flexibility may be recommended adjunct therapies for this population.

## Figures and Tables

**Table 1:** Bivariate Pearson correlation matrix of measures at baseline.

	ASI	AAQ	API	DASS-SF D	DASS-SF A	DASS-SF S	PDI
ASI	1	.551^*∗∗*^	.123	.318^*∗∗*^	.317^*∗∗*^	.402^*∗∗*^	.155^*∗*^
AAQ		1	.133^*∗*^	.398^*∗∗*^	.399^*∗∗*^	.398^*∗∗*^	.172^*∗∗*^
API			1	.250^*∗∗*^	.274^*∗∗*^	.257^*∗∗*^	.518^*∗∗*^
DASS-SF D				1	.846^*∗∗*^	.789^*∗∗*^	.316^*∗∗*^
DASS-SF A					1	.816^*∗∗*^	.352^*∗∗*^
DASS-SF S						1	.339^*∗∗*^
PDI							1

AAQ: Action and Acceptance Questionnaire; ASI: Anxiety Sensitivity Index; API: average pain intensity; DASS-SF D: Depression Anxiety Stress Scales-Short Form, Depression subscale; DASS-SF A: Depression Anxiety Stress Scales-Short Form, Anxiety subscale; DASS-SF S: Depression Anxiety Stress Scales Short Form, Stress subscale; PDI: Pain Disability Index; ^*∗∗*^ *p* < 0.01; ^*∗*^ *p* < 0.05.

**Table 2:** Demographic and clinical characteristics of study population and participants subdivided into the five-cluster subgroups.

	Study population	Cluster 1	Cluster 2	Cluster 3	Cluster 4	Cluster 5	*p* (among clusters)
*N*	229	38	26	59	63	43	
Mean age (SD)	45.6 (11.5)	44.7 (12.6)	45.3 (9.2)	46.8 (13.4)	45.2 (11.1)	45.2 (9.8)	>0.05
Sex (M %)	35.8	24.3	38.5	40.7	39.7	32.6	>0.05
Relationship status (%)							
Single	12.8	13.5	8.0	12.7	13.3	14.3	>0.05
Married or in a serious relationship	73.1	67.6	80.0	69.1	76.7	73.8	
Divorced, separated, and widowed	14.2	18.9	12.0	18.2	10.0	11.9	
Pain duration (%)							
Less than 5 years	47.4	47.4	34.6	46.6	44.4	60.5	>0.05
5 to 10 years	37.3	36.8	53.8	32.8	39.7	30.2	
Greater than 10 years	15.4	15.8	11.5	20.7	15.9	9.3	
ASI (SD)	22.0 (12.4)	42.1 (9.0)	30.4 (5.0)	21.8 (5.6)	14.7 (4.6)	10.0 (6.0)	<0.001
AAQ (SD)	32.1 (7.8)	41.1 (5.6)	28.7 (3.9)	37.6 (3.3)	30.6 (2.5)	20.9 (4.0)	<0.001

AAQ: Acceptance and Action Questionnaire; ASI: Anxiety Sensitivity Index.

**Table 3:** Mean values (standard deviation) in pain intensity, mood, and disability among the cluster subgroups at 1 year follow-up.

	Cluster 1	Cluster 2	Cluster 3	Cluster 4	Cluster 5	*F*	*p*
Average pain intensity	5.9 (2.0)	5.5 (1.9)	6.0 (1.8)	5.9 (2.1)	5.4 (2.0)	1.45	0.22
DASS total	30.0 (13.7)	21.2 (13.0)	26.3 (11.8)	20.3 (10.6)	18.7 (12.3)	11.81	**0.001**
DASS depression	9.6 (4.8)	6.0 (4.4)	8.4 (4.7)	6.6 (4.1)	5.7 (4.1)	10.76	**0.001**
DASS anxiety	10.3 (4.6)	7.6 (4.5)	9.5 (4.1)	7.1 (4.1)	6.7 (4.2)	10.62	**0.001**
DASS stress	10.0 (5.0)	7.3 (4.4)	8.2 (4.2)	6.6 (3.1)	6.3 (4.6)	12.28	**0.001**
PDI	37.8 (12.0)	33.6 (13.8)	37.7 (16.6)	38.0 (14.1)	34.0 (18.9)	4.83	**0.001**

Bolded values denote *p* < 0.05. DASS: Depression Anxiety Stress Scales; PDI: Pain Disability Index.

**Table 4:** Post hoc analysis in mood and disability among clusters subgroups.

	Cluster 1	*p*	Cluster 2	*p*	Cluster 3	*p*	Cluster 4	*p*	Cluster 5	*p*
	Mean difference		Mean difference		Mean difference		Mean difference		Mean difference	
DASS total										
1			8.5 (2.5)	**0.008**	4.1 (2.1)	>0.05	10.1 (2.1)	**<0.001**	13.3 (2.2)	**<0.001**
2					−4.4 (2.4)	>0.05	1.6 (2.3)	>0.05	4.8 (2.5)	>0.05
3							6.0 (1.8)	**0.011**	9.2 (2.0)	**<0.001**
4									3.2 (2.0)	>0.05

DASS depression										
1			3.4 (1.0)	**0.005**	1.3 (0.8)	>0.05	3.4 (0.8)	**<0.001**	4.8 (0.8)	**<0.001**
2					−2.0 (0.9)	>0.05	0.03 (0.9)	>0.05	1.4 (0.9)	>0.05
3							2.1 (0.7)	**0.02**	3.5 (0.8)	**<0.001**
4									1.34 (0.7)	>0.05

DASS anxiety										
1			2.8 (0.9)	**0.021**	0.9 (0.7)	>0.05	3.0 (0.7)	**0.001**	4.4 (0.8)	**<0.001**
2					−1.8 (0.8)	>0.05	0.2 (0.8)	>0.05	1.6 (0.9)	>0.05
3							2.1 (0.7)	**0.013**	3.5 (0.7)	**<0.001**
4									1.4 (0.7)	>0.05

DASS stress										
1			2.6 (0.9)	**0.025**	1.8 (0.7)	>0.05	3.8 (0.7)	**<0.001**	4.7 (0.8)	**<0.001**
2					−0.8 (0.8)	>0.05	1.2 (0.8)	>0.05	2.1 (0.8)	>0.05
3							2.0 (0.6)	**0.01**	2.9 (0.7)	**<0.001**
4									0.8 (0.7)	>0.5

PDI										
1			6.3 (2.8)	>0.05	2.4 (2.3)	>0.05	5.3 (2.2)	>0.05	9.6 (2.4)	**<0.001**
2					−3.9 (2.6)	>0.05	−1.0 (2.5)	>0.05	3.3 (2.7)	>0.05
3							2.9 (2.0)	>0.05	7.2 (2.2)	**0.009**
4									4.3 (2.2)	>0.05

Bolded values denote *p* < 0.05. DASS: Depression Anxiety Stress Scales; PDI: Pain Disability Index.
